# A Multi-Fidelity Model for Simulations and Sensitivity Analysis of Piezoelectric Inkjet Printheads

**DOI:** 10.3390/mi12091038

**Published:** 2021-08-29

**Authors:** Vinh-Tan Nguyen, Jason Yu Chuan Leong, Satoshi Watanabe, Toshimitsu Morooka, Takayuki Shimizu

**Affiliations:** 1 Institute of High Performance Computing, 1 Fusionopolis Way, #16-16 Connexis, Singapore 138632, Singapore; jason-leong@ihpc.a-star.edu.sg; 2 Seiko Holdings Corp., 8, Nakase 1-chome, Mihama-ku, Chiba-shi 261-8507, Chiba, Japan; satoshi.watanabe@seiko.co.jp; 3 SII Printek Inc., 563, Takatsukashinden, Matsudo-shi 270-2222, Chiba, Japan; toshimitsu.morooka@sii.co.jp (T.M.); taka.shimizu@sii.co.jp (T.S.)

**Keywords:** inkjet printing, droplet-on-demand, piezoelectric actuators, computational fluid dynamics, fluid structure interactions, lumped element method, surrogate based optimisation

## Abstract

The ink drop generation process in piezoelectric droplet-on-demand devices is a complex multiphysics process. A fully resolved simulation of such a system involves a coupled fluid–structure interaction approach employing both computational fluid dynamics (CFD) and computational structural mechanics (CSM) models; thus, it is computationally expensive for engineering design and analysis. In this work, a simplified lumped element model (LEM) is proposed for the simulation of piezoelectric inkjet printheads using the analogy of equivalent electrical circuits. The model’s parameters are computed from three-dimensional fluid and structural simulations, taking into account the detailed geometrical features of the inkjet printhead. Inherently, this multifidelity LEM approach is much faster in simulations of the whole inkjet printhead, while it ably captures fundamental electro-mechanical coupling effects. The approach is validated with experimental data for an existing commercial inkjet printhead with good agreement in droplet speed prediction and frequency responses. The sensitivity analysis of droplet generation conducted for the variation of ink channel geometrical parameters shows the importance of different design variables on the performance of inkjet printheads. It further illustrates the effectiveness of the proposed approach in practical engineering usage.

## 1. Introduction

The piezoelectric drop-on-demand inkjet print method consists of an ink channel which deforms due to piezoelectric actuation at the ink channel walls. Upon application of a voltage, the deformation of the actuator causes the ink channel volume to increase. Deactivating the applied voltage causes the ink channel to contract and ejects a droplet at the ink channel nozzle [1]. Precise control of the actuation voltage pulse allows for fine control of the ejected droplet’s volume and velocity.

In commercial applications, ink channels are arranged together in a print head where hundreds to thousands of individual ink channels work in unison. It is recognised that, to achieve greater print speed by increasing the ink drop jetting frequency and print resolution by packing more ink channels closer together, there arises the issue of residual vibration and cross-talk between ink channels [2,3,4]. Understanding of the physics embedded in the inkjet head, as well as their interactions, has enabled engineers to design more robust and efficient devices for droplet-on-demand printing.

Traditionally, the design of inkjet heads is based on engineering rules and carried out through a series of design cycles in which many prototypes were made and tested to analyse their performance. This process of trial and error is time-consuming, involving a large number of iterations and extensive human resources.

Inkjet print head design is a difficult problem to model because of the physics involved: electrical, mechanical, and fluid mechanics [5]. The piezoelectric actuators used to deform the ink channel walls can be modelled as a coupled electro-mechanical system using the finite element method. Some opensource solvers can do this [6]. A fluid–structure interaction (FSI) approach involves the coupling of the electro-mechanical domain with the fluid domain. This approach is usually performed using the partitioned method approach where both domains share a common interface [7,8]. Fully resolved FSI simulations may become too computationally prohibitive to perform for optimum design studies so there is an interest in reduced-order models.

Instead of a fully resolved model in both domains, it is possible to model the electro-mechanical system using the lumped element method (LEM) while still maintaining the high fidelity, fully resolved, 3D Navier-Stokes solver (CFD) for the fluid domain [5]. The LEM here describes the electro-mechanical domain as an equivalent electrical circuit with lumped parameters of analogous electrical components (resistors, capacitors, and inductors) to describe the electrical and mechanical properties. The equivalent circuit approach is further refined to include both domains to study the behaviour of inkjet printheads [9,10,11].

Fully modelling both domains as an equivalent circuit greatly reduces the complexity of the problem. The use of an equivalent circuit in the analysis of an ink channel draws many parallels to its usage in microfluidic circuit analysis [12,13,14]. It is also possible to link all domains with proper transformer expressions to facilitate the modelling of multidisciplinary domains, and to enable the co-simulation of all linked domains as a single system in a compact circuit simulator such as SPICE (Simulation Program with Integrated Circuit Emphasis) [15]. The accuracy of any equivalent circuit models depends on the evaluation of its lumped parameters for various elements in the circuit. In practice, simplification of physics and geometrical complexity is normally used to obtain lumped parameters [9,10]. This could lead to large errors and over-simplification when using the LEM approach for prediction of system performance. Here, three-dimensional computational models of different physical components of the printhead are used to quantify the lumped parameters for assessment of printheads under varying design configurations. To the best of the authors’ knowledge, this is the first time that such an approach is proposed and coupled with LEM for simulations of inkjet heads.

The rest of the paper is organized as follows. The next section provides a brief description of three-dimensional models used in this work for the simulation of printhead channels. In Section 3, the lumped element model of the printhead channel is discussed with the emphasis on a three-dimensional simulation-based approach for the estimation of LEM parameters. The proposed methodology is then applied for a commercial printhead channel in Section 4. Results obtained from LEM are validated against prediction from a three-dimensional fluid–structure interaction approach. Section 5 of the paper focuses on the optimization of printheads, channels, and their operational conditions for a desired droplet size. The section includes some discussion on the optimal convergence and posterior error estimation of the optimum configuration.

## 2. Three-Dimensional Model of Inkjet Head Channel

**Figure 1:**
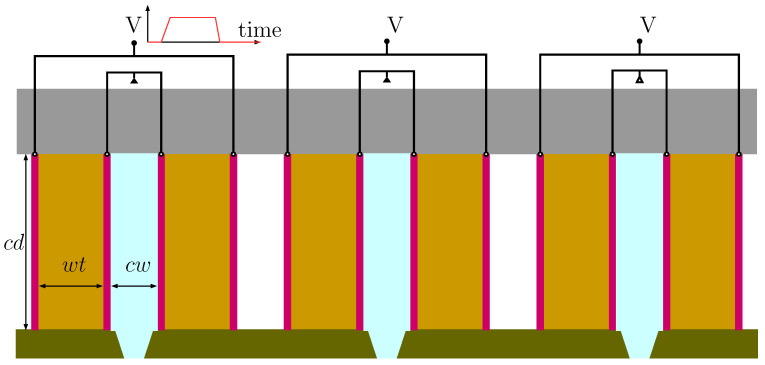
Sketch of RC1536 inkchannel including the piezoelectric actuator wall with attached electrodes, cover plates, nozzle plate, and ink chamber. Here, cd,cw,wt are the channel depth, channel width, and wall thickness of the channel. The image is not to scale.

**Figure 2:**
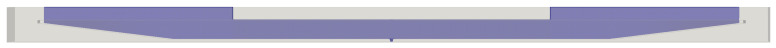
A three-dimensional view of a typical inkjet head channel including actuator structure (in light grey shaded volume) and ink volume (in dark blue color).

In this work, we considered a series of recirculation printheads manufactured by Seiko Instruments Incorporated (SII) [16]. The printheads as shown in Figure 1 feature a recirculation chamber design with isolated channel nozzles for fast and effective printing on various substrates with different types of inks. Figure 2 shows a three-dimensional view of the RC1536 printhead channel where the ink chamber is designed for better recirculation of the ink, and droplet ejection is driven by piezoelectric actuator walls.

Typically, the process of piezoelectric inkjet ejection is a multiphysics coupled problem in which the electric field is coupled to the mechanical deformations of the piezoelectric actuator. The electrically-actuated channel then interacts with fluid (ink) flow dynamics for droplet ejection. Simulations of this process require two essential models. Firstly, a computational model for electromechanical actuation is used to simulate the effects of the electric field on the deformation of the piezoelectric walls. Next, a flow–structure interaction model is considered for simulation of the droplet generation. Effectively, these two models are tightly coupled via deformation of the wall, driving the printing process. A brief description of the two models is presented in the following section.

### 2.1. Electromechanical Coupled Model of Channel Walls

The dynamic response of a piezoelectric continuum of volume Ω bounded by surface Γ=∂Ω are governed by momentum conservation equations as follows:

(1) $$\begin{array}{c} \rho {\ddot{u}}_{i}=\sigma_{ij,j}+\rho f_{i}^{B}, \end{array}$$

while Gauss’s law for electric fields in a dielectric is given as

(2) $$D_{i,i}=0$$

Here $\sigma_{ij}$ and $D_{i}$ are the components of the Cauchy stress tensor and electric displacement vector. $\rho ,f_{i}^{B}$ are density and body force, respectively. The displacement vector equation is the Gauss law written in the absence of free charges. The subscripts ${\left(\right)}_{i}$ and ${\left(\right)}_{ij}$ represents the *i*th and (i,j)th component of a vector and a matrix, respectively. The symbols $\ddot{\left(\right)}$, and ${\left(\right)}_{,i}$ represent $\frac{\partial^{2}\left(\right)}{\partial t^{2}}$ and $\frac{\partial ()}{\partial x_{i}}$, respectively. For the coupled electromechanical problem, the constitutive equations are as follows:

(3) $$\begin{array}{cc} \; & \sigma_{ij}=C_{ijkl}\varepsilon_{kl}-e_{kij}E_{k}, \end{array}$$

(4) $$\begin{array}{cc} \; & D_{i}=e^{ikl}\varepsilon_{kl}+\epsilon_{ik}E_{k}, \end{array}$$

where $C_{ijkl},\;\epsilon_{kl},\;and\;e_{kij}$ are elastic, dielectric material, and piezoelectric constants, respectively. The Cauchy strain tensor $\varepsilon_{kl}$ is defined as follows:

(5) $$\varepsilon_{kl}=\frac{1}{2}\left(u_{k,l}+u_{l,k}\right)$$

and the electric field vector component, with an assumption of negligible magnetic effects, is irrotational and can be represented in terms of electric potential, ϕ:

(6) $$\varepsilon_{ijk}E_{i}E_{j}=0\;=>\;E_{i}=-\phi_{,i},$$

where $\varepsilon_{ijk}$ represents the Levi-Civita symbol.

The electro-mechanical coupled system is discretized using the finite element approach as described in the earlier work [6]. The model was an extension of the traditional nodal-based FEM formulation, with the additional unknown for electrical potential at every node to take into account the coupling effects. The model has been well validated with different piezoelectric structures. One can refer to the earlier work [6] for more details about the model implementation and its applications.

### 2.2. Ink Flow Model

Ink flow in the channel is modeled as an isothermal compressible fluid governed by the Navier-Stokes equations describing conservation of mass and momentum as follows:

(7) $$\begin{array}{cc} \frac{\partial \rho }{\partial t}+\nabla \left(\rho u\right) & =0 \end{array}$$

(8) $$\begin{array}{cc} \frac{\partial \rho u}{\partial t}+\rho \left(u\nabla \right)u & =-\nabla p+\nabla \left(\mu \nabla u\right)-gx\nabla \rho +f_{b} \end{array}$$

In the above equations, ρ, *p*, and **u** are the density, dynamic pressure, and velocity of the mixture. It is also noticed that **g** denotes the gravitational acceleration and $f_{b}$ is the body force acting on the fluid. The ink is considered a weakly compressible liquid, simply described by the equation of state of barotropic function as follows:

(9) $$\psi =\frac{d\rho }{dp}=\frac{\rho }{K},$$

where *K* is the compressibility constant. The above equation of state is linearised to describe the relationship between density and pressure as follows:

(10) $$\rho =\rho_{0}+\psi \left(p-p_{0}\right),$$

where $\rho_{0}$ and $p_{0}$ are the reference density and pressure values satisfying $\rho_{0}=\rho \left(p_{0}\right)$. Despite operating at low Mach number in most inkjet devices, the ink compressibility effect is significant due to the high impulse impact of the piezoelectric actuators on fluids. It is believed that the weakly isothermal compressible model is sufficiently suitable to model ink flow dynamics in the channel.

The above set of governing equations for isothermal compressible Navier-Stokes flows are discretized using cell-centred finite volume approach implemented in OpenFOAM [17,18].

## 3. Equivalent Circuit Model of Inkjet Head Channel

**Figure 3:**
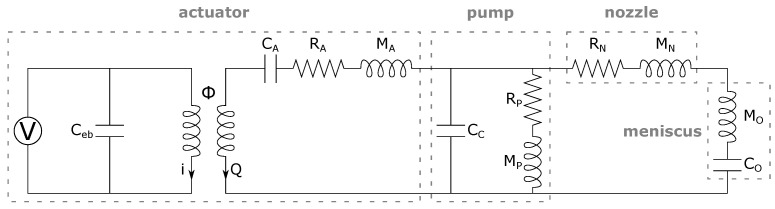
Equivalent circuit model of inkjet actuator and channel.

For the given inkjet head as shown in Figure 2, it is commonly possible to employ a lumped element model (LEM) as an alternative approach to model such a complex coupled system. In LEM, each mechanical component is represented by an equivalent electrical element depending on its function. These are analogous to the components and quantities in the electrical circuit and fluidic circuit. In particular, the electrical current (*i*) and voltage are equivalent to the volumetric flow rate (*Q*) and pressure in the fluidic circuit, respectively. Similarly, electrical capacitance, resistance, and inductance are analogous to fluidic capacitance, inertance, and resistance, respectively [12,15]. Earlier work on LEM has been used to model synthetic jet actuators [19,20] and was later adopted to model inkjet channels [9,10,11]. In this work, the full ink channel and the piezoelectric actuator are represented by an equivalent circuit as illustrated in Figure 3.

### 3.1. Equivalent Circuit Model

In this equivalent circuit, the actuator is modelled by the two main fluidic and electrical circuits responsible for the piezoelectric actuation. Following earlier work [20], the electrical circuit consists of the voltage source, V, and the blocked electrical capacitance, $C_{eb}$ of the piezoelectric element in the actuator. The blocked electrical capacitance is defined as follows:

(11) $$C_{eb}=C_{ef}\left(1-\kappa^{2}\right)$$

where $C_{ef}$ is the free electrical capacitance and κ is the electro-acoustic coupling factor, defined by

(12) $$\kappa^{2}=\frac{d_{A}^{2}}{C_{ef}C_{A}}$$

The short circuit acoustic compliance of the actuator ($C_{A}$), and the effective acoustic piezoelectric coefficient ($d_{A}$) are analogous to the displaced volume when only pressure or voltage is applied, respectively, and are defined as follows:

(13) $$d_{A}=\frac{v}{V}{|}_{P=0}=\frac{1}{V}\int u\left(x\right)ds$$

(14) $$C_{A}=\frac{v}{P}{|}_{V=0}=\frac{1}{P}\int u\left(x\right)ds$$

where *v* is the displaced volume evaluated by the integral, ∫u(x)ds, *V* is the applied voltage and *P* is the applied pressure. The remaining lumped element parameters for the actuator are the inertance, $M_{A}$, and resistance, $R_{A}$ defined as follows:

(15) $$M_{A}=\frac{\rho_{s}}{v}\int {\dot{u}}^{2}\left(x\right)ds$$

(16) $$R_{A}=2\xi \sqrt{\frac{M_{A}}{C_{A}}}$$

where ξ is the damping ratio of the actuator. Note that the coupling factor, ϕ, represented by the transformer component in Figure 3, is needed to couple the electrical and fluidic circuits. It is a ratio of the effective acoustic piezoelectric coefficient and the short circuit acoustic compliance of the actuator [19]:

(17) $$\phi =\frac{d_{A}}{C_{A}}$$

The ink channel capacitance, $C_{C}$, in the pump is defined as follows:

(18) $$C_{C}=\frac{v_{C}}{\rho_{o}c_{o}^{2}}$$

where $v_{C}$ is the channel volume, $\rho_{o}$ is the ink density, and $c_{o}$ is the speed of sound in ink. $R_{P}$ and $R_{N}$, the pump and nozzle hydraulic resistances, respectively, are defined as follows:

(19) $$R_{i}={\left(\frac{\Delta P}{Q}\right)}_{i},i=P,N$$

ΔP here is the pressure across the component of interest and *Q* is the volumetric flow rate. The pump and nozzle inertances are quantified by equating the integral of the distributed kinetic energy and the lumped kinetic energy [20]:

(20) $$\frac{1}{2}\rho \int \dot{u}\left(v\right)dv=\frac{1}{2}MQ^{2}$$

Finally, at the nozzle exit, the meniscus is formed under some applied pressure. The effect of the meniscus on holding a certain volume of ink is represented by a capacitance defined as [10,11]

(21) $$C_{o}=\frac{\pi r^{4}}{3\sigma },$$

where σ is the surface tension of the ink and *r* is the radius of meniscus curvature. Here it is assumed that the meniscus has the shape of half of a sphere; thus, the meniscus curvature is the same as the radius of the nozzle exit.

### 3.2. 3D Simulation-Based LEM Parameters

The LEM parameters described in the previous section are required to perform simulations of the equivalent circuit model for various inkjet printhead designs. These parameters depend on several factors including ink properties, material properties, and geometric design variations. Given a design configuration, the estimation of the LEM parameters can be done in several ways to evaluate the performance of the inkjet head. The most commonly used approach is to apply analytical formulations to canonical flows such as pipe or channel flows to approximate the lumped parameters [20]. Instead of using simple analytical models as in the earlier work [9,10], three-dimensional high fidelity models are proposed in this work to evaluate the LEM parameters to take into account the effects of changes in designs more effectively.

**Figure 4:**
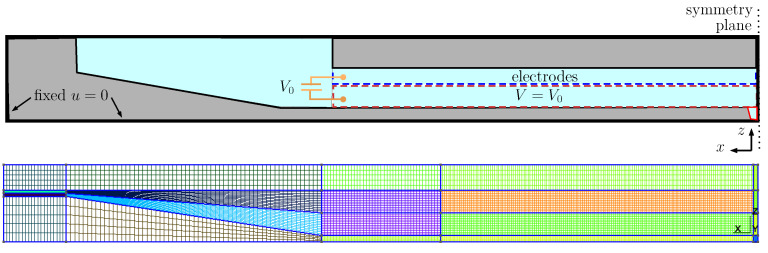
(**Top**) computational domain and boundary condition for the solid part of the inkjet channel. (**Bottom**) a typical block-structured mesh for calculating the actuator lumped parameters. The domain is partitioned uniformly in the central actuating region with a specific grid resolution Δx,Δy,Δz in three directions, x,y,z, respectively.

**Figure 5:**
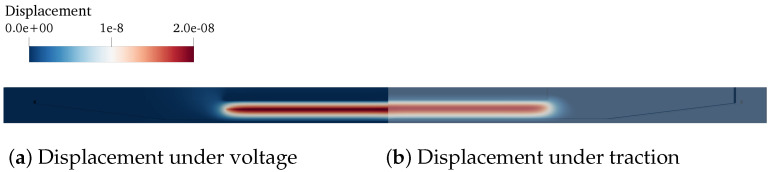
Displacement obtained from FEM analysis of the ink channel wall under (**a**) voltage and (**b**) pressure loading. The load is applied at the actuation boundary patch, as shown in Figure 4.

In particular, a finite element model of the inkjet head channel is constructed to obtain the actuator-associated LEM parameters ($C_{A}$, $d_{A}$, $M_{A}$, and $R_{A}$). A typical computational domain of an inkjet channel is shown in Figure 4, where a block structure grid is generated using Gmsh [21]. For a given ink head design, the displaced volume (∫u(x)ds) was calculated for two separate load cases with different boundary conditions of prescribed traction and voltage. Figure 5 shows a typical result of displacement on the channel walls under traction and voltage loading. This displacement field is then used to compute the corresponding LEM parameters following the formulations in the earlier section. The FEM analysis is capable of taking into account the effects of geometrical and physical parameters on the lumped elements, thus enabling more design-specific characterization of the inkjet head channel.

**Figure 6:**
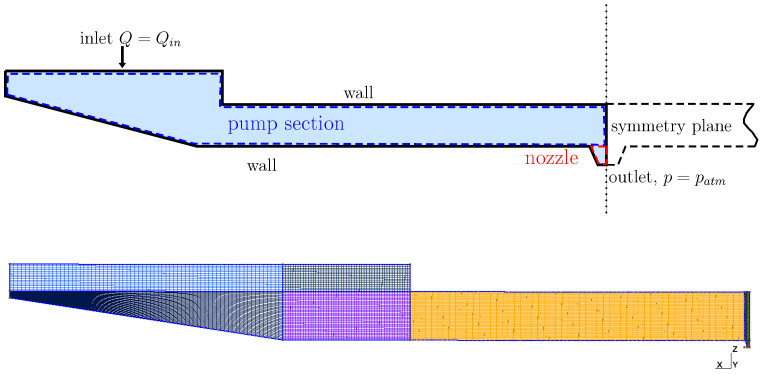
(**Top**) Computational domain and boundary condition for fluid section of the ink channel. The fluid domain is partitioned into two sections, namely, pump and nozzle, to quantify the LEM parameters $M_{P}$, $M_{A}$, $R_{P}$, and $R_{N}$. (**Bottom**) A typical mesh for simulation where the middle pump section (in yellow) is uniformly meshed with resolution of Δx, Δy, and Δz.

**Figure 7:**
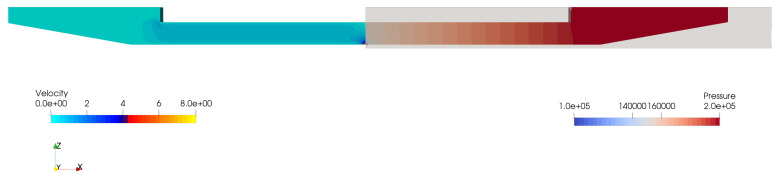
Pressure and velocity in fluid volume of the channel computed using 3D CFD model to obtain fluidic LEM parameters.

Similarly, the single-phase fluid solver described above was used to obtain fluidic LEM parameters ($C_{C}$, $R_{N}$, $R_{P}$, $M_{N}$, and $M_{P}$). It is straightforward for the computation of capacitance, $C_{C}$, where its dependence on the channel volume, $V_{C}$, can be directly obtained from its geometry. $R_{N}$ and $R_{P}$ are obtained by performing a steady-state flow simulation to calculate the pressure drop, ΔP, and flow rate, *Q*, across the nozzle and pump, respectively, as seen in Figure 6. Here, the flow rate is obtained by applying a pressure drop between the inlet patch and the nozzle outlet. Typical pressure and velocity in the channel are shown in Figure 7, where it can be seen that the pressure drop has caused a net flow passing through the nozzle. Quantifying flow rates across various sections of the channel provides information for the calculation of resistance and inertance of the channel.

It is worth noting that all the meshes used in these 3D simulations are block-structured for the ease of parametrising the structural and fluid geometry. Using the block-structured mesh ensures consistent mesh count and quality for efficient pre- and post-processing. In this work, the geometric parameters of interest, such as ink channel depth, ink channel width, wall thickness, and channel length, can be defined as variables in the pre-processing stage. Finally, the whole process of design parametric exploration and computation LEM parameters is integrated and automated. It should be noted that this framework is quite flexible and can be modified to vary other input parameters, such as structural and ink properties.

**Figure 8:**
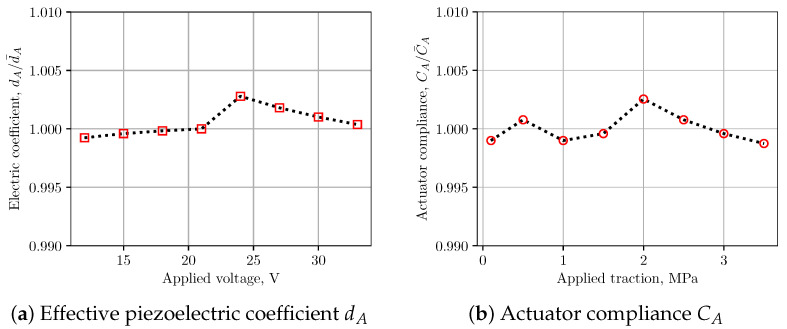
Variation of channel LEM parameters with applied traction and voltage. It is noted that piezoelectric coefficient and acoustic compliance are not very sensitive to applied loading conditions.

Using the above approach, performing structural and fluid analysis with 3D simulations on two separate domain is able to provide lumped parameters of the ink channel for subsequent LEM predictions. A grid sensitivity study was carried out to verify the calculation of the LEM parameters. Details of the grids used for computation of LEM parameters are provided in Figure 4 and Figure 6, where the computational domains are partitioned into block-structured meshes. It is noted that, in both fluid and structural domains, the region containing the nozzle is uniformly partitioned with the highest resolution in three directions to be able to resolve the nozzle dimensions. Table 1 and Table 2 present the grid convergence index (GCI) [22] for LEM parameters of the channel with changes in grid resolution. The GCI was computed for different parameters obtained from simulations on successively refined grids. It can be seen that at grid level G2 the GCI is below 1% for most of the parameters. In Figure 8, the lumped parameters were shown for variation in loading conditions of voltage and pressure. It can be seen that those parameters are less sensitive to the loads; therefore, a representative value of pressure (1.5 $\times \;10^{5}$ Pa) and voltage (20 V) can be conveniently chosen for the subsequent computation of lumped parameters.

## 4. Numerical Validation of the Models

In this section, validation of the multifidelity LEM approach with the experiment and available literature data is presented for two problems: a synthetic jet device and an industrial print head.

### 4.1. LEM Modelling of Piezoelectric-Driven Synthetic Jets

**Figure 9:**
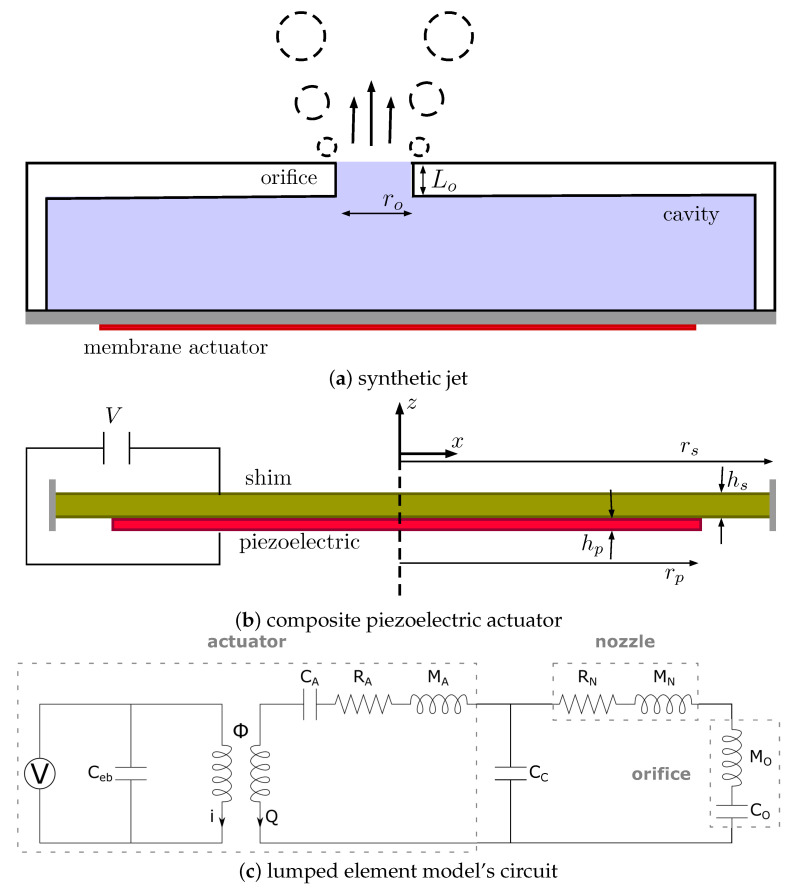
Details on the set-up of the synthetic jet case, including the construction of a typical device (**a**) including the composite actuator (**b**) and the lumped element model (**c**).

The proposed multifidelity approach is first applied for the simulation of piezoelectric-driven synthetic jets [20,23,24]. Figure 9 shows a typical set-up of a synthetic jet device comprising of a piezoelectric actuator and a cavity with an orifice for jetting. When actuating with an input voltage signal, the composite plate of an piezoelectric layer and a metal shim oscillates and pushes the air through the orifice, also known as a zero-net mass flux actuator. The pieoelectric-driven diaphram is characterized by the shim plate of radius $r_{s}$ and thickness $h_{s}$, and the piezoelectric layer of radius $r_{p}$ and thickness $h_{p}$. The orifice section is described by the radius $r_{o}$ and the neck length $L_{o}$ connected to a cavity of volume $v_{C}$. Table 3 lists the dimensions of those parameters for three synthetic jet configurations reported in the earlier work [20] which are used for validation in this work. Among the three designs, the SJ0 model only comprises the actuator component, which was used to validate the LEM model for its structural response.

**Figure 10:**
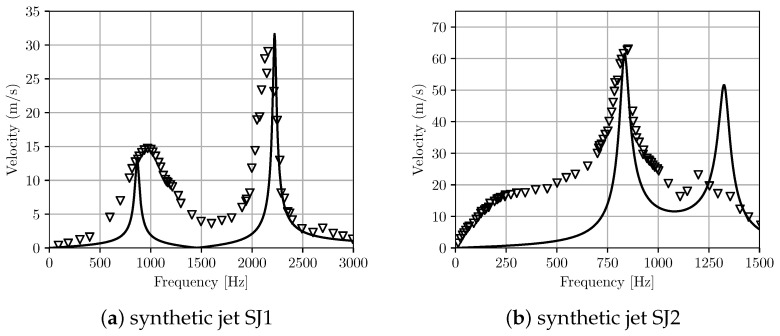
Velocity at the orifice with frequency for two synthetic jet set-ups. Comparison between the present LEM prediction with experiment [20]. The lines are the numerical prediction using the proposed LEM approach and the triangle symbols are experimental data.

Here, we used the same lumped element model (LEM) as in the earlier work [20] for the device. Table 4 presents values of the lumped parameters obtained from the present 3D simulation-based approach in comparison with the analytical one for different actuator configurations. The calculated lumped parameters of the effective acoustic piezoelectric coefficient ($d_{A}$) and the mass (or inertance, $M_{A}$) of the diaphragm are in good agreement with results obtained from the analytical approach; however, there is a larger discrepancy for short-circuit acoustic compliance, $C_{A}$. It is noted that these parameters are calculated in the analytical approach based on the linear composite plate theory. Using that approach, for the estimation of the actuator’s acoustic compliance, it is assumed that the piezoelectric plate effect is negligible compared to the metal shim; thus, the compliance of the diaphragm is modelled as that of a homogeneous clamped circular plate. As cautioned in the earlier work [19,20], this assumption could lead to an inaccurate calculation of the acoustic compliance and the current results reaffirm that. The natural frequency of the actuator was also computed from the lumped parameters as $f_{D}=1/2\pi \sqrt{1/M_{A}C_{A}}$ and shown in Table 4, with reasonable agreement between the analytical and present 3D simulation-based estimation. Figure 10 shows the maximum velocity across the orifice versus frequency for two synthetic jet configurations listed in Table 3. Both configurations display a typical two resonance frequency response. Results from the current LEM agree with the experimental data for the maximum velocity at peak frequencies, especially for the SJ1 configuration. In the other set-up (SJ2), the model predicts a higher velocity at the second peak frequency compared to the earlier experiment where the response was strongly damped. Away from the resonant frequencies, the present model predicts lower velocity compared to the experimental data.

### 4.2. Prediction of LEM for Print Heads

**Figure 11:**
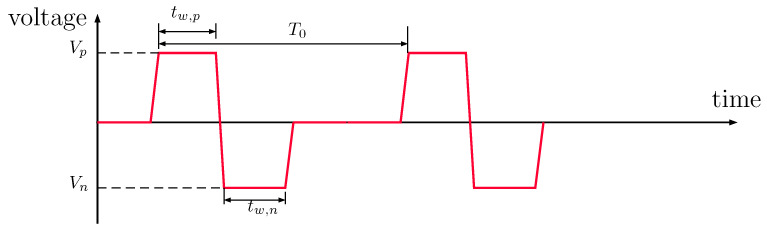
A general square pulse shape for controlling droplet generation with RC1536. The pulse includes negative and positive applied voltage ($V_{n},V_{p}$), pulse width ($t_{w,n},t_{w,p}$), and a period $T_{0}$.

For further validation of the proposed approach, simulations were conducted for RC1536 printhead [16] at a typical operating condition recommended by the manufacturer. The input voltage is in a square form shown in Figure 11 with a positive and negative pulse and the amplitude voltage of $V_{ref}=20$ V. The channel was first modelled using the LEM approach and subsequently by a three-dimensional fluid–structure interaction approach for comparison. Using the 3D simulation-based lumped parameters obtained in the earlier section, performance of the channel can be predicted by running transient analysis of the equivalent circuit. In the FSI simulation, responses of the electromechanical actuator under the applied voltage are coupled to the fluid domain by deforming the channel walls, thus generating acoustic waves to drive the ink flow in the channel. The pressure waves from ink flows acting on the channel walls are taken into account by two-way coupled approach. For more details on the FSI simulations, one can refer to the earlier work [25].

**Figure 12:**
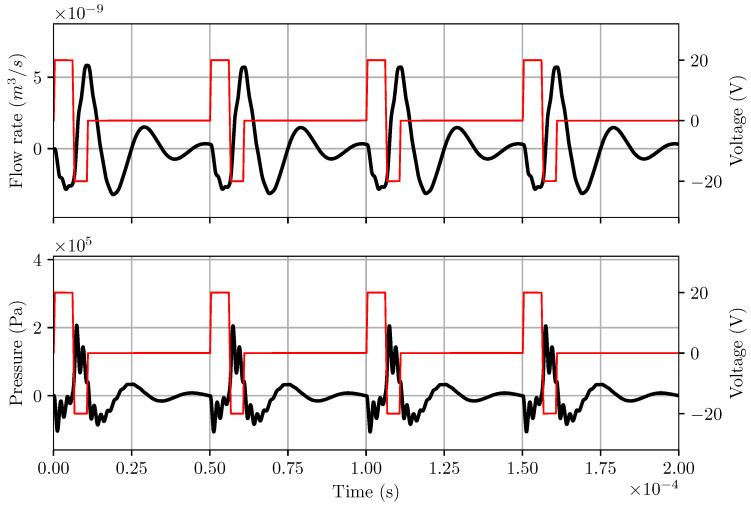
Output from LEM for RC1536 inkjet head channel with dimensioned given by SII at a reference operating condition of $V_{ref}=20$ V and pulsewidth of 8.0 μs. Here the red thin lines are voltage signal applied at the actuator and black thick lines are flowrate and pressure profiles.

**Figure 13:**
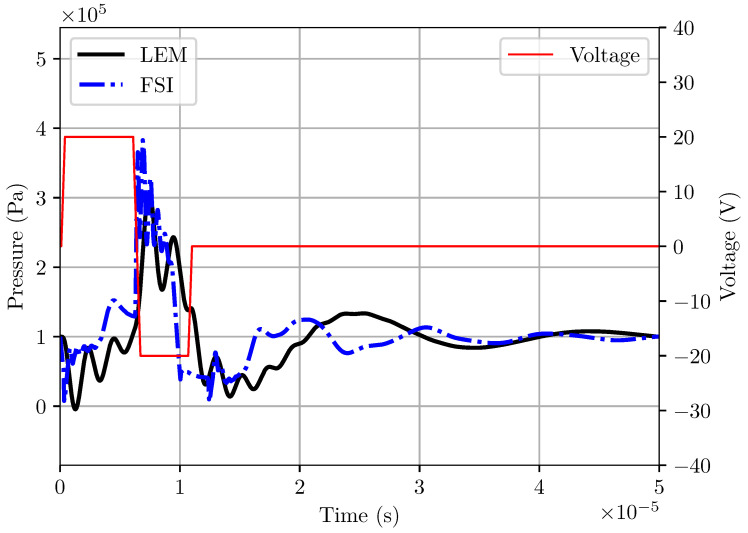
Comparison of pressure obtained from LEM with a 3D fluid–structure interaction model for RC1536 head.

The flowrate and pressure at the entrance of the ink channel’s nozzle are extracted from the LEM simulation and plotted in Figure 12. The pressure profile clearly demonstrates that the LEM is able to capture the pull and push action of the actuator under oscillating applied voltage, resulting in fluctuations of flowrate across the nozzle. Figure 13 shows a comparison of the pressure signal at the nozzle obtained from LEM and two-way coupled FSI approaches. It is observed that the LEM result of the pressure profile is comparable with the 3D FSI results. However, there are some discrepancies of the pressure profile between LEM and FSI predictions during the activation stage. Notable differences in the amplitude of pressure oscillations immediately after pulling and pushing phases demonstrated a lack of damping effects in the LEM simulation. It can be explained by the limitation of the LEM approach in taking into account the effects of meniscus shapes and oscillations of the ink circulation in the channel.

In the next test, a positive–negative pulse shape, as shown in Figure 11, was applied with a variation of pulse width and pulse height, scaled with controlled values of period ($T_{c}$) and voltage ($V_{c}$). The controlled period of the pulse is based on the characteristic frequency of the channel and calculated as $T_{c}=2\pi \sqrt{M_{A}\left(C_{A}+C_{C}\right)}$, while the controlled voltage is selected from the specified operation of the integrated circuit unit. First, 3D simulations were conducted for both fluid volume and actuation structure to determine the lump element model (LEM) parameters. The resulting LEM parameters were fed into the equivalent circuit to simulate the response of the channel under different voltage inputs. Finally, droplet characteristics (velocity and volume) were recorded and compared with the experimental data.

**Figure 14:**
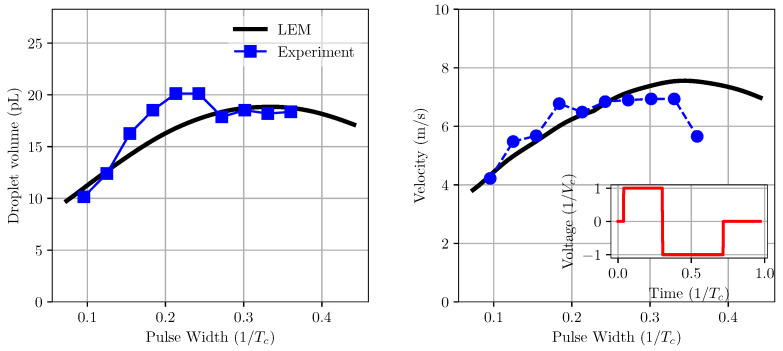
Variation of droplet volume and velocity with pulse width in comparison with experiment measurement. The on-pulse condition predicted from the model is in good agreement with experimental data of about 0.3$T_{c}$.

From LEM simulations, the droplet velocity is directly obtained by the flowrate profiles at the nozzle outlet and its cross sectional area. To obtain droplet volume, the flow rate is integrated over the period of droplet ejection [26]. In the case of the present inkjet head under the negative–positive pulse shape, the start of ejection is normally after the pushing action, resulting in positive volume flow rates. Figure 14 shows the variation in droplet velocity and volume with the changes in the positive pulse width. In this case, the negative pulse width and pulse height were kept constant at 0.4$T_{c}$ and $V_{c}$, respectively. It can be seen that droplet velocity and volume increase with an increase in positive pulse width until about 0.3–0.35$T_{c}$, where the velocity and volume reach their maximum values. When the pulse width increases further, the droplet velocity drops while its volume remains unchanged. A similar trend is observed from experimental data for both droplet velocity and volume. However, the on-pulse condition (the pulse width at which the velocity is maximal) is slightly different between numerical and experimental data. The results showed a fairly good agreement between the model and experiment for velocity, especially for pulse widths of less than 0.3$T_{c}$. It is also observed that, while the trend obtained from the model for droplet volume with pulse width is comparatively good in comparison with the experiment, there is a marked difference between the values of droplet volume. This could be due to a couple of reasons. First, the droplet volume data in the model is derived from mass flow rate prediction without taking into account the droplet formation at the nozzle tip. Secondly, there is a large uncertainty in the experimental data, which is clearly shown in the fluctuation of the measurements.

**Figure 15:**
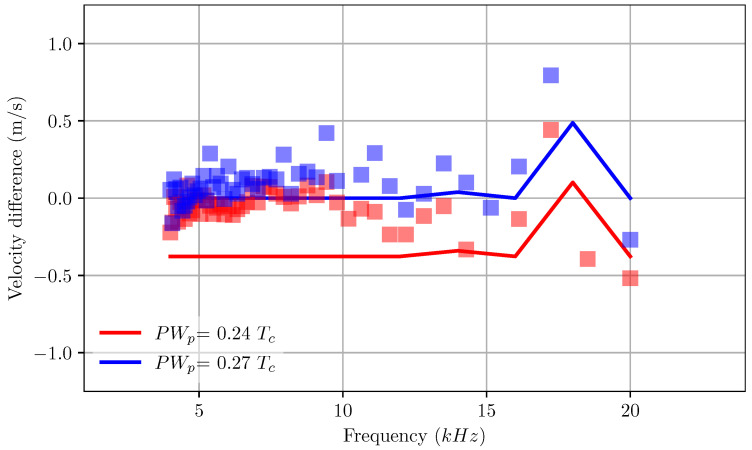
Variation in droplet velocity with applied frequency with changes in pulse width. The velocity is measured as the difference from droplet velocity at a pulse width of $0.27T_{c}$ and frequency of 5 kHz. The lines are a numerical prediction of droplet velocity with changes in pulse frequency, symbols are experimental measurements.

It is widely known that in practice the droplet speed varies with the pulse frequency. A good design of the channel will likely ensure a low fluctuation of speed for a wide range of frequencies. Figure 15 shows the variation in droplet speed of RC1536 as a function of the applied frequency. From the experiment data, it is noted that there is a large fluctuation of droplet velocity with the frequency. This fluctuation is even apparent and significant at the low-frequency range. However, results from the current model predict larger fluctuations only at higher frequencies, beyond 15 KHz for a range of pulse width. This current result demonstrates the uncertainty and accuracy of measurements used in the experiment.

**Figure 16:**
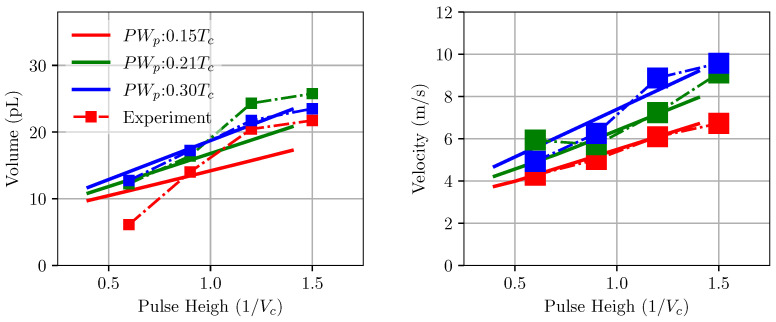
Behaviour of droplet volume and velocity with changes in pulse height (voltage) for the RC1536 channel obtained from experiment and model. The model prediction is in good agreement with the experiment measurements.

Figure 16 shows the variation in droplet velocity and volume with changes in positive pulse height and pulse width. It can be seen that both velocity and volume increase when a stronger voltage is applied. This is reasonable since a stronger voltage will result in large wall displacement, thus pushing more ink through the channel at a faster speed. While the model predicts the trend well, there are large differences in the value of velocity and volume in comparison with the experiment. At lower positive pulse width, the prediction of droplet velocity is in good agreement with the experiment. This is consistent with the earlier comparison in Figure 14. A similar trend is also observed for velocity and volume when changing the negative pulse height. It is, however, noted that the drop velocity remains the same at a given pulse height for varying negative pulse width. Again, this result is consistent with the earlier one from Figure 7, showing constant velocity for a large range of negative pulse widths. The differences between model and experiment can be partially attributed to the uncertainty in measurement.

## 5. Analysis of Inkjet Head Performance Using LEM

The predictability of the LEM circuit depends on the accuracy of its LEM parameters obtained from 3D simulations. For any specific design or configuration of the inkjet head channel, it is necessary to compute a new set of lumped parameters to characterize its droplet generation performance. Here we considered a variation in geometrical design parameters of the channel such as channel width, depth, wall thickness, and electrode height from the baseline configuration of the commercial RC1536 inkjet head. In this section, the sensitivity analysis was carried out for LEM parameters and inkjet head’s performance for changes in channel geometrical design variables.

### 5.1. Sensitivity Analysis of LEM Parameters

**Figure 17:**
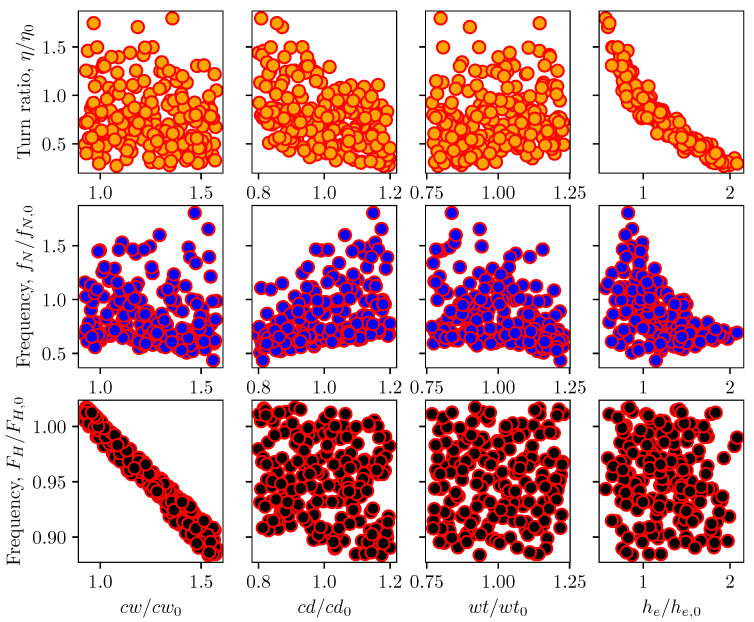
Scatter plot of inkjet channel frequency with geometrical parameters of channel width, depth, wall thickness, and electrode height. The parameters are normalized with the base channel of the existing RC1536 inkjet head.

**Figure 18:**
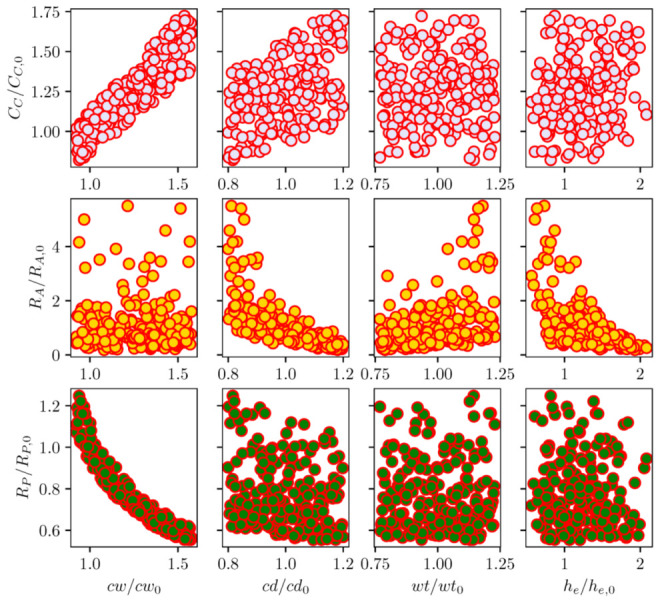
Scatter plot of circuit element coefficients (resistance and capacitance) with geometrical parameters of channel width, depth, wall thickness and electrode height. The parameters are normalized with the base channel of the existing RC1536 inkjet head.

A sensitivity analysis was performed for lumped element coefficients with the variation in channel design parameters. Here, the channel width, depth, wall thickness, and its electrode height were perturbed from the baseline configuration of the existing RC1536 head. Figure 17 and Figure 18 depict the scattering of channel actuation parameters and ink chamber resistance lumped coefficients with changes in channel geometry for 200 samples. From the scatter plots, it can be seen that the channel width has more influence on the Helmholtz frequency of the actuator, as well as the channel capacitance and pump resistance, due to its direct control of the ink volume in the channel. The channel width is seen to have a more significant influence on the resistance of the actuator, and thus on its natural frequency. Overall, most of the LEM parameters show widely scattered patterns with geometrical designs. This indicates the coupled effects of design parameters on the channel performance.

It is hard to quantify the sensitivity of LEM parameters to geometrical variables based on the above scatter plots. In sensitivity analysis, it is common to use the variance-based decomposition [27] as a global sensitivity method to evaluate the uncertainty in the output response apportioned to the uncertainty in input variables. In a nutshell, this approach computes sensitivity indices to quantify the influence of input parameters on the variance in output responses. The indices include first and second-order sensitivity $S_{i},i=1,2$ and the total effect index $S_{T}$. The main effect first and second-order indices measure contributions to the variance in the output by individual inputs and the coupled effects of two inputs, respectively. The total effect index measures the contribution of an individual variable as well as high-order interactions between input parameters to the variance in the output. The indices can be computed as follows:

(22) $$S_{i}=\frac{{\mathrm{Var}}_{x_{i}}(E\left(y|x_{i}\right)}{\mathrm{Var}(y)},\;S_{T}=\frac{\mathrm{Var}\left(y\right)-\mathrm{Var}(E\left(y|x_{-i}\right)}{\mathrm{Var}(y)}.$$

Here $y=f(x_{i})$ is the response output as a function of input parameters $x_{i}$ and $x_{-i}=\left\{x_{1},x2,\ldots ,x_{i-1},x_{i+1},\ldots ,x_{N}\right\}$. The computation of the above indices is based on the variance decomposition approach, evaluating the multidimensional integrals of decomposed functions approximating partial variances of the output. There exist several methods for the computation of sensitivity indices such as Sobol [28], Morris and FAST [29]. In this work, the opensource package Dakota [27] is used to perform the sensitivity analysis.

**Figure 19:**
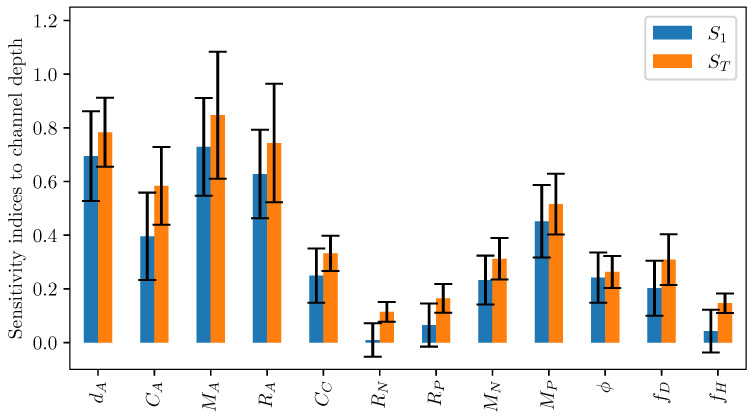
First order ($S_{1}$) and total sensitivity ($S_{T}$) indices of different lumped element parameters to channel depth. The error bar indicates 95% confidence interval.

**Figure 20:**
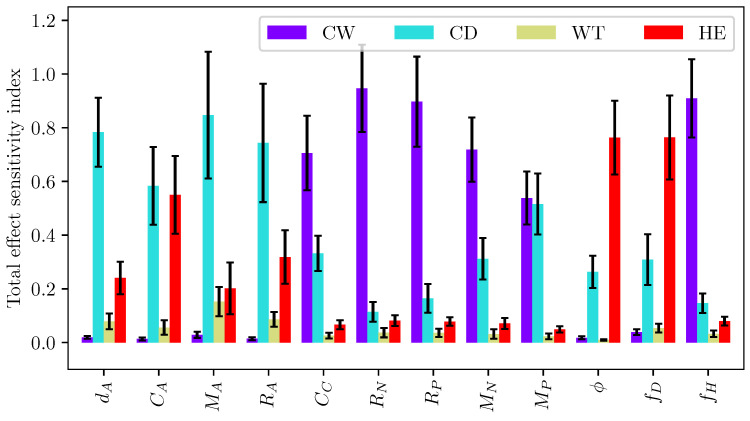
Total sensitivity ($S_{T}$) index of different lumped element parameters with geometrical parameters. The error bar indicates 95% confidence interval.

Figure 19 plots the first-order and total effect sensitivity index of channel width on the LEM coefficients. It can be seen that the channel contributes more significantly to the uncertainty of the actuator’s LEM parameters as compared to the pump section’s coefficients. It is also reasonably understood that its contribution to the pump innertance is greater than other coefficients related to the pump section. The total effect sensitivity index is consistently higher than the first-order index across all output coefficients as it takes into account higher-order interactions of the channel width variable with other design parameters. Figure 20 shows the total effect sensitivity index for all input variables of the channel’s LEM parameters. The contribution of channel depth to the pump section is clearly seen in this graph. It is noted that the wall thickness has a much lower contribution to all LEM parameters in comparison with other input variables, indicating its lesser importance in the design of the inkjet head channel.

### 5.2. Analysis of Inkjet Head Performance

**Figure 21:**
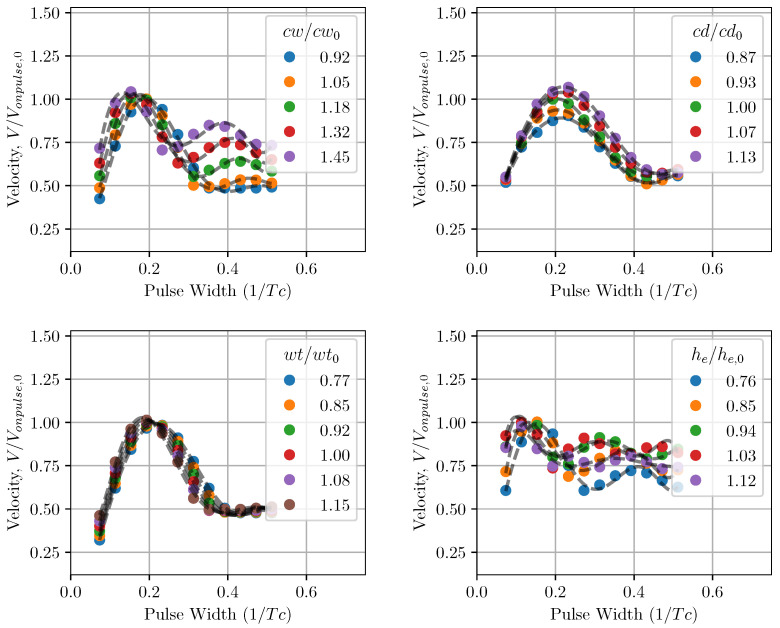
Pulse characteristics of droplet velocity in the inkjet head with changes in the channel’s geometrical parameters. Note the effect of the dimensions of the inkjet channel on on-pulse condition.

**Figure 22:**
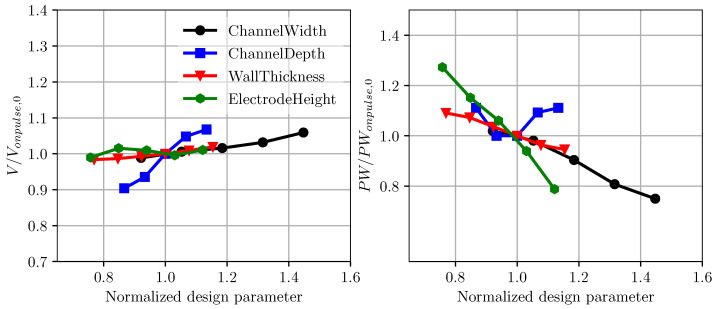
Onpulse velocity and pulse width with changes in channel dimensions. The quantities are normalized with the baseline condition.

The performance of the inkjet head channel is next investigated by varying the channel geometrical parameters. In Figure 21 the droplet velocity prediction using LEM is plotted against changing pulse width. In this case, the response of the inkjet head is measured for a single pulse input with the reference applied voltage of $V_{ref}$ as in the previous validation study. For a given configuration of channel geometry, the droplet velocity varies with applied pulse width and reaches a maximum value, normally referred to as on-pulse condition. Here, the droplet velocity is normalized with the on-pulse droplet velocity for the baseline RC1536 channel. The on-pulse velocity is plotted against the channel geometrical parameters in Figure 22. Generally, the channel’s on-pulse velocity increases with the increase in channel width, channel depth, and wall thickness, while it remains invariant with the electrode height. In comparison with all the parameters, the rate of increase in the droplet velocity is highest with channel depth, demonstrating its important influence on droplet velocity. Figure 22 also shows the variation in on-pulse pulse width with the channel design parameters. While the on-pulse pulse width generally reduces with channel width, wall thickness, and electrode height, its variation with channel depth is more parabolic, with clear minimum values of the pulse width. The minimum on-pulse pulse width is observed to be very close to the baseline RC1536 design, showing the optimal power usage consideration in the current ink channel design.

**Figure 23:**
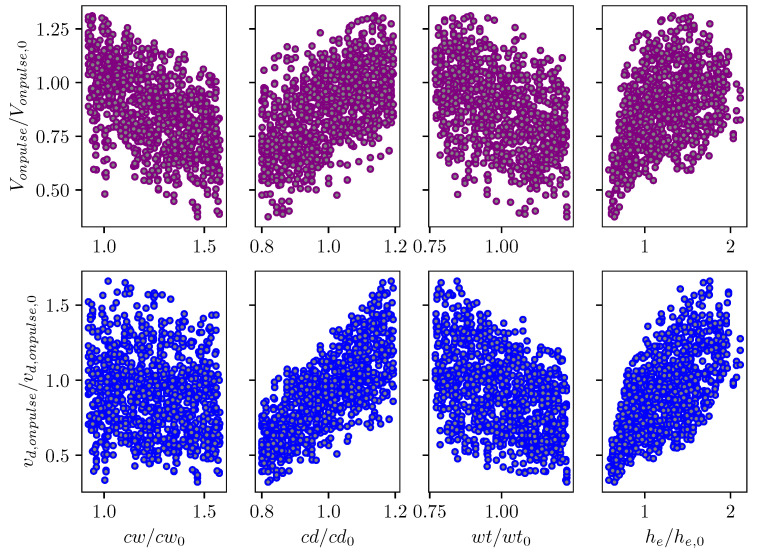
Scatter plot of normalized droplet volume and velocity with changes in inkjet channel geometrical parameters.

**Figure 24:**
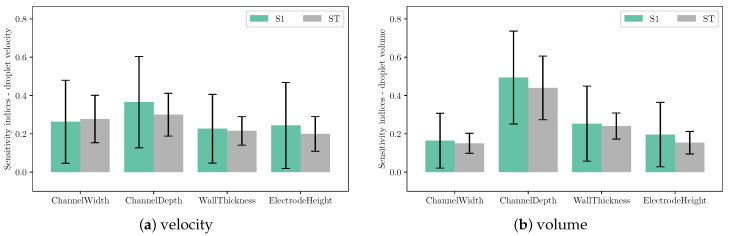
First order ($S_{1}$) and total sensitivity ($S_{T}$) indices of (**a**) droplet velocity and (**b**) volume to channel design parameters. The error bar indicates 95% confidence interval.

Sensitivity analysis was carried out for droplet velocity and volume with variations in channel geometrical parameters. In this Sobol’s analysis, the input parameters are sampled using Saltelli’s sampling approach [29] and simulations were performed for each of those sampled configuration to obtain the droplet velocity and volume. Figure 23 depicts the scattering of output velocity and volume with respect to the channel parameters. It is observed that there is a strong interaction between design parameters and droplet generation, with no apparent dominant influence of a single input. This is clearly shown in the sensitivity indices for droplet speed and volume in Figure 24. It can be seen that all design parameters show the same level of influence on droplet velocity. With low first order and total effect index, the channel width does not contribute much to the variance in droplet volume, while the influence of channel depth on droplet volume is more significant compared to other parameters.

## 6. Conclusions

In this work, a lumped element model (LEM) for an inkjet head was developed to simulate its droplet generation in inkjet printing. The model parameters were calculated from the output of three-dimensional fluid and structure simulations, thus making it a model specific to an inkjet head, while running significantly faster than the high fidelity 3D simulations. The 3D based fast LEM was validated against the experimental data from an existing commercial head, RC1536 [16]. The LEM is capable of capturing multiphysics effects in the droplet generation process, resulting in good agreement with data on characteristics of the inkjet head. Compared to the high fidelity multiphysics fluid-structure interaction (FSI) simulations, the LEM also showed encouraging results in pressure wave prediction at the nozzle inlet. The model was then applied for sensitivity analysis of channel design variations on inkjet head performance. Through the analysis of variance, channel width and depth were identified as important parameters in the design of the channel, while the wall thickness is much less influential in inkjet printing performance. It was also noted that the current design of the baseline RC1536 channel was optimal in terms of on-pulse conditions with a specified droplet velocity. The current development and analysis will provide a good platform for future work on the design and optimization of inkjet head printers.

## Figures and Tables

**Table 1:** Grid convergence index for the strcutural LEM parameters showing good convergence obtained from the simulations. Here Δx, Δy, and Δz are the grid resolution in x,y,z directions in the central region of the computational domain. The nozzle region has a uniform mesh of resolution Δz in all directions. The grid size is normalized with the nozzle radius, *r*.

	Mesh				Grid Convergence Index (%)			
	r/Δx	r/Δy	r/Δz	Cells	$d_{A}$	$C_{A}$	$M_{A}$	$R_{A}$
G0	1.25	2.50	5.0	42,420	-	-	-	-
G1	1.875	3.75	7.5	82,264	7.56	0.50	1.86	0.77
G2	2.50	5.00	10.0	136,992	2.87	0.21	0.67	0.15
G3	3.75	7.50	15.0	306,308	0.82	0.11	0.16	0.06

**Table 2:** Grid convergence index for the fluidic LEM parameters. Symbols are the same as in Table 1.

	Mesh				Grid Convergence Index (%)			
	r/Δx	r/Δy	r/Δz	Cells	$R_{P}$	$R_{N}$	$M_{P}$	$M_{N}$
G0	2.0	8.0	2.0	35,416	-	-	-	-
G1	3.0	12.0	3.0	96,312	6.50	1.02	1.39	1.07
G2	4.0	16.0	4.0	196,576	2.24	0.42	0.57	0.57
G3	6.0	24.0	6.0	596,928	0.52	0.12	0.16	0.23

**Table 3:** Geometrical specifications of three synthetic jet configurations extracted from [20]. Configuration SJ0 is corresponding to the stand-alone actuation test; SJ1 and SJ2 are case 1 and case 2 in the cited work, respectively.

Configuration	SJ0	SJ1	SJ2
Shim radius, $r_{s}$ [mm]	11.5	11.5	18.5
Shim thickness, $h_{s}$ [mm]	0.201	0.150	0.100
Piezo-plate radius, $r_{p}$ [mm]	10.0	10.0	12.5
Piezo plate thickness, $h_{p}$ [mm]	0.232	0.08	0.11
Cavity volume, $v_{C}$ [mm${}^{3}$]	-	2.50	5.50
Orifice radius, $r_{o}$ [mm]	-	0.825	0.420
Orifice length, $L_{o}$ [mm]	-	1.65	0.84

**Table 4:** LEM parameters for different synthetic jet configurations using the 3D simulation based approach and analytical estimation [20].

		$C_{A}\times 10^{13}$	$d_{A}\times 10^{11}$	$M_{A}$	$\phi_{a}$	κ	$f_{D}$
		(s${}^{2}$m${}^{3}$/kg)	(m${}^{3}$/V)	(kg/m${}^{4}$)	(Pa/V)	-	(Hz)
SJ0	Analytical [20]	1.491	2.077	13,538.0	139.3	0.117	3542.4
	3D based, present	0.998	1.977	12,956.1	198.0	0.254	4425.1
SJ1	Analytical [20]	7.391	5.528	7670.0	74.79	-	2114.0
	3D based, present	5.188	5.285	7003.6	101.87	0.297	2640.0
